# Temporal dynamic effects of cortical resilience proteins on trajectories of cognitive aging

**DOI:** 10.1002/alz.089162

**Published:** 2025-01-09

**Authors:** Andrea R Zammit, Tianhao Wang, Lei Yu, Shahram Oveisgharan, Philip L. De Jager, Vladislav A Petyuk, David A. Bennett, Aron S Buchman

**Affiliations:** ^1^ Rush Alzheimer's Disease Center, Chicago, IL USA; ^2^ Department of Neurology, Columbia University Medical Center, New York, NY USA; ^3^ Biological Sciences Division, Pacific Northwest National Laboratory, Richland, WA USA; ^4^ Rush Alzheimer's Disease Center, Rush University Medical Center, Chicago, IL USA

## Abstract

**Background:**

In prior work we identified cortical resilience proteins associated with the linear trajectories of cognitive decline independent of the effects of neuropathology. Some of these proteins were associated with slower and some with faster cognitive decline. We tested the hypothesis that the temporal onset and duration of effects of cortical resilience proteins associated with cognitive trajectories may vary in aging adults.

**Method:**

We used data from 1,088 older participants (31% males, mean age at death =89.7, SD = 6.4; mean education = 16.3 years, SD = 3.6) from two clinical‐pathologic studies. Participants completed a battery of 19 cognitive tests annually, underwent neuropathologic examination, and had targeted proteomic analysis performed in the dorsal lateral prefrontal cortex. To identify cortical resilience proteins, we first ran linear mixed effects models with cognitive decline as outcome, and the proteins as predictors, controlling for the effects of neuropathologic indices. To estimate the temporal effect of the identified resilience proteins on cognitive decline, we then ran functional mixed effects models, which allow i) individuals to have heterogeneous (linear and nonlinear) patterns of cognitive decline, and ii) the influence of predictors to vary flexibly over time.

**Result:**

We identified 40 cortical resilience proteins which linear and nonlinear effects on cognitive decline spanned the entire studied trajectory of up to 25 years of follow‐up. We found that the effects of 17 proteins were significantly nonlinear: while 10 were associated with higher resilience, 7 were associated with lower resilience. The onset of effects of these proteins showed that i) the effects of higher resilience (slower rate of decline) proteins start up to 23 years before death, while the effects of the lower resilience (faster rate of decline) proteins are restricted within the last 7 years of life.

**Conclusion:**

Multiple cortical proteins may underlie the resilience afforded by varied lifestyles and behaviors. Yet, the temporal window during which different proteins may provide cognitive resilience may vary. Different analytic techniques may be needed to elucidate the molecular mechanisms underlying cognitive resilience and their critical temporal window during later life.

